# Case Report: Uniportal Video-Assisted Thoracoscopic Parenchymal Sparing Secondary Carinal Resection and Reconstruction for the Treatment of Tracheobronchial Mucoepidermoid Carcinoma

**DOI:** 10.3389/fsurg.2021.823281

**Published:** 2022-01-18

**Authors:** Yan Hu, Xiaofeng Chen, Siying Ren, Chao Zeng, Li Wang, Peng Xiao, Fenglei Yu, Wenliang Liu

**Affiliations:** ^1^Department of Thoracic Surgery, The Second Xiangya Hospital of Central South University, Changsha, China; ^2^Hunan Key Laboratory of Early Diagnosis and Precision Treatment of Lung Cancer, The Second Xiangya Hospital of Central South University, Changsha, China; ^3^Department of Anesthesia, The Second Xiangya Hospital of Central South University, Changsha, China; ^4^Department of Respiratory and Critical Care Medicine, The Second Xiangya Hospital of Central South University, Changsha, China; ^5^Department of Cardiothoracic Surgery, The Third Xiangya Hospital of Central South University, Changsha, China

**Keywords:** uniportal, video-assisted thoracoscopic surgery, parenchymal sparing procedure, secondary carinal reconstruction, tracheobronchial mucoepidermoid carcinoma

## Abstract

Surgical resection is currently the mainstay of treatment for tracheobronchial mucoepidermoid carcinoma (TMEC). The parenchymal sparing secondary carinal resection and reconstruction for TMEC under the uniportal thoracoscopic approach has seldomly been reported in the literature. Here, we report a case of a 42-year-old male patient complaining of the incidental finding of a 1.5 × 1.2 cm neoplasm at the opening of the right bronchus intermedius and a 5.1 × 3.1 cm patchy lesion located at the left upper lobe by chest CT scans in February 2021. This patient successively underwent fiberoptic bronchoscopic biopsy of the bronchial neoplasm and CT-guided biopsy of the left upper lobe lesion. Pathological examination confirmed the diagnosis of the endobronchial mass in the right bronchus intermedius as low-grade mucoepidermoid carcinoma and left upper lobe lesion as tuberculosis. This patient successfully underwent uniportal thoracoscopic parenchymal sparing tumor resection, reconstruction of the secondary carina and lymphadenectomy at our center. Intraoperative frozen section showed no residual cancer at any bronchial stumps. Postoperative pathology indicated that no metastases were seen in any of the resected lymph nodes. The patient recovered well after surgery. He received a 9-month course of anti-tuberculosis treatment postoperatively. He did not complain of any special discomfort and there was no local recurrence at the 9-month postoperative follow-up. Although technical demanding, this case highlights that uniportal video-assisted thoracoscopic parenchymal sparing secondary carinal resection and reconstruction for TMEC is safe and feasible with the preservation of lung function and excellent outcomes.

## Introduction

Tracheobronchial mucoepidermoid carcinoma (TMEC) is a rare malignant tumor of the lung originating from the seromucous submucosal glands of the lower respiratory tract, accounting for ~0.1–0.2% of all lung malignancies (1). TMEC can be classified into low-grade and high-grade subtypes, based on its histological appearance, nuclear division ability, cellular atypia, local invasion and necrosis. Low-grade TMEC has a more favorable prognosis than high-grade TMEC, and complete surgical resection alone is sufficient, whereas high-grade TMEC often requires adjuvant therapy after surgery (2).

Bronchoplasty is the treatment of choice for low-grade malignant tumors of the bronchus, including TMEC (3). In recent years, parenchymal sparing procedures (PSP) have been developed to minimize the volume of parenchymal resection. There are a few reports in the literature indicating that PSP achieves excellent functional and oncological outcomes in selected cases of low-grade endobronchial neoplasms (4).

Here, we report the first case of uniportal video-assisted thoracoscopic parenchymal sparing right secondary carinal resection and reconstruction for the treatment of TMEC.

## Case Presentation

**Figure 1:**
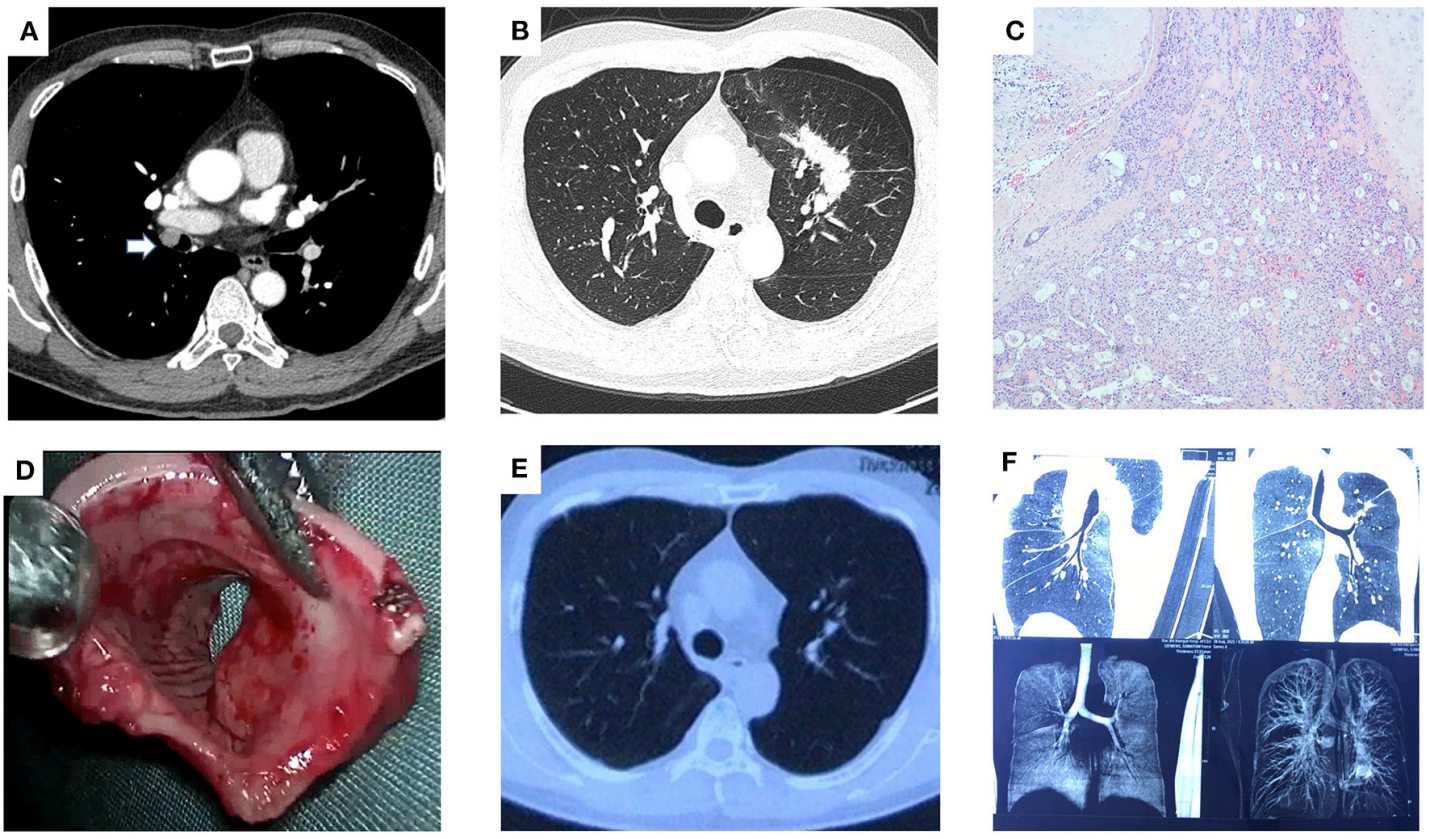
Uniportal VATS secondary carinal resection and reconstruction for tracheobronchial mucoepidermoid carcinoma. Contrast-enhanced CT of the chest showed a 1.5 × 1.2 cm endobronchial nodule of the right bronchus intermedius (see arrow, **A**) and a 5.1 × 3.1 cm patchy lesion centrally located at the left upper lobe **(B). (C)** Postoperative pathology confirmed the diagnosis of the well-differentiated mucinous epidermoid carcinoma. **(D)** Gross appearance of the endobronchial mass. **(E)** Contrast-enhanced CT of the chest at the 6-month postoperative follow-up showed resolution of tuberculosis lesion in the left upper lobe. **(F)** CT bronchography at the 6-month postoperative follow-up showed no bronchial stenosis of the right lung.

A 42-year-old asymptomatic male patient received thoracic CT scans on a routine health examination at a local hospital in February 2021 with an incidental finding of a 1.5 × 1.2 cm endobronchial nodule of the right bronchus intermedius and a 5.1 × 3.1 cm patchy lesion centrally located at the left upper lobe (Figure 1). The patient underwent fiberoptic bronchoscopy, which showed a neoplasm with an unsmooth surface at the opening of the right bronchus intermedius with normal distant bronchial tree. Transbronchial biopsy of the neoplasm was suggestive of low-grade mucinous epidermoid carcinoma. The patient further underwent PET/CT scans, which revealed weak FDG uptake in the endobronchial nodule and high uptake in the left upper lobe lesion. To discern the nature of the left upper lobe lesion, the patient underwent a CT-guided percutaneous lung biopsy. Pathological examination revealed a chronic granulomatous inflammatory disease, suggesting the diagnosis of tuberculosis. Due to the specific location of the endobronchial tumor, the patient was recommended by the treating physician at the local hospital to receive bronchoscopic cryoablation over surgical resection.

**Figure 2:**
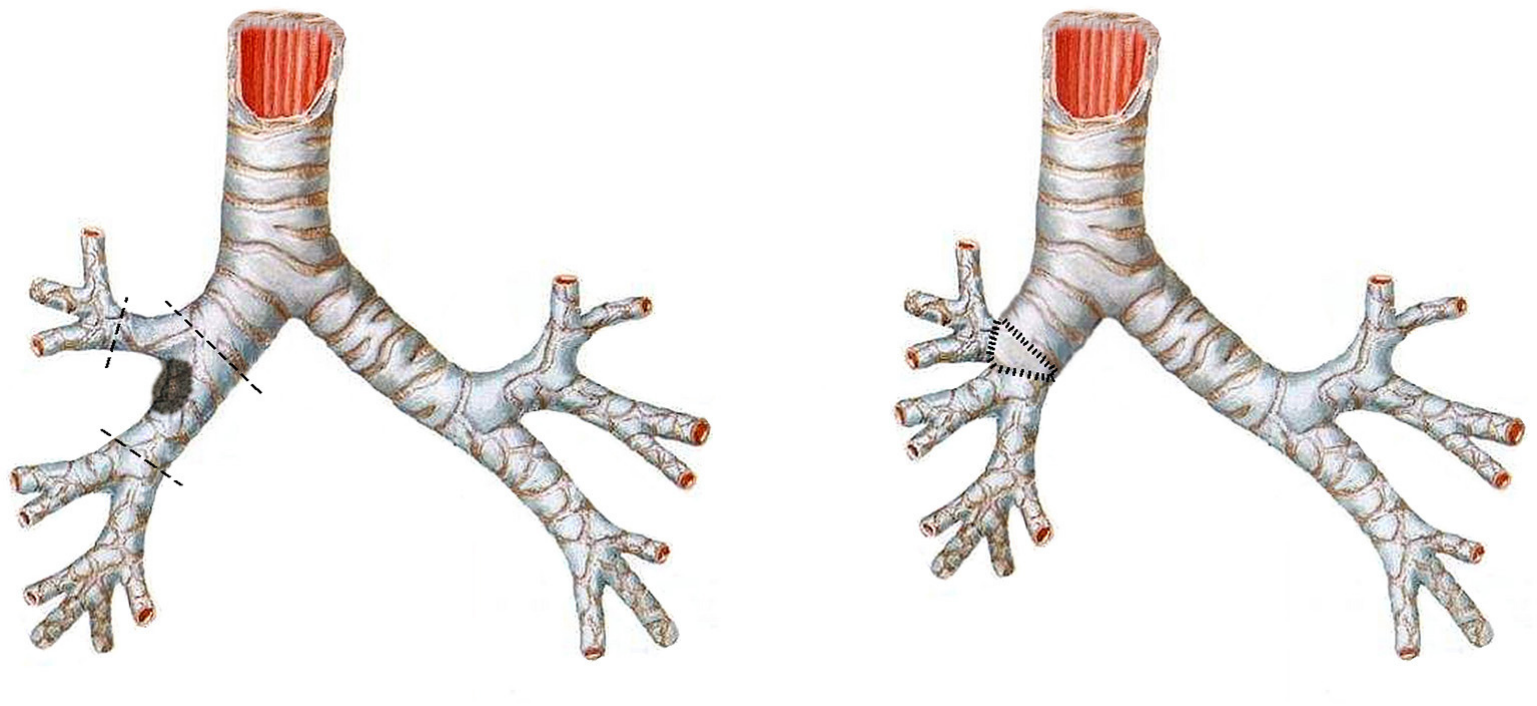
The schematic illustration of right secondary carinal resection and reconstruction.

Because of concerns that the tumor might recur with bronchoscopic therapy, the patient was referred to our department for seeking surgical resection. The results of preoperative routine blood biochemistry were normal. Pulmonary function test showed mild obstructive ventilation dysfunction, with FEV1 being 2.38L and FEV1%PRED being 76%. Considering the patient's 30-year history of bronchial asthma (manifested as wheezing sounds in both lungs) and the fact that lung parenchymal resection might lead to decreased quality of life, the patient underwent a single-port thoracoscopic tumor resection, reconstruction of the secondary carina and systemic lymph node dissection (including stations 2, 4, 7, 9, 10, 11, 12) in March 2021 (Figure 2). The patient was placed in a left lateral decubitus position. After the intubation of the double-lumen tube, single lung ventilation of the contralateral lung was performed under general anesthesia. The utility incision was a 3-cm incision at the 4th intercostal space along the anterior axillary line. After the careful dissection of the right main bronchus, the right upper bronchus and the right bronchus intermedius, the tumor at the opening of the right bronchus intermedius was resected (see Supplementary Video 1). The stumps were trimmed and sent for intraoperative frozen section, which showed no residual cancer at any bronchial stumps. Then, the anastomosis procedure was initiated. First, the lateral walls of the right upper bronchus and the right bronchus intermedius were sutured continuously to join up with a 3-0 Prolene (one thread with two needles). Second, two another 3-0 Prolene (both sutured with one stitch in the bronchi nearby) were tied with the two ends of the first 3-0 Prolene into a knot outside of the bronchi, respectively. Subsequently, a circumferential and continuous suture of the right main bronchus with the right upper bronchus and the right bronchus intermedius was performed via these two 3-0 Prolene and a knot was tied outside of the bronchi. Last, the anterior wall of secondary carina was reinforced with interrupted sutures using a 3-0 Monocryl (Y316, one thread with one needle) and the anterior mediastinal fatty tissue was freed to cover the anastomosis with another 3-0 Monocryl. There were no air leakage from the anastomotic area after puffing of the lungs with the addition of warm saline to the chest cavity. The operation lasted 200 mins totally. Postoperative pathology showed solid adenoid arrangement of tumor cells and focal invasion of bronchial cartilage, consistent with a well-differentiated mucinous epidermoid carcinoma. No lymph node metastasis was observed. The patient recovered well after surgery and was discharged on postoperative day 3. He receive anti-tuberculosis treatment for 9 months postoperatively and did not complain of any specific discomfort at the 9-month postoperative follow-up, with no local recurrence observed.

## Discussion

TMEC was first reported by Smetana et al. and originates from the seromucous submucosal glands in the lower respiratory tract and has the gross appearance of an exophytic endobronchial circumscribed mass (1). The symptoms of TMEC are often non-specific and depend on the location of the tumor (2). Central tumors can present with bronchial obstruction, often in the form of cough, dyspnoea, wheezing and haemoptysis. Currently, surgical resection is the treatment of choice (2). For the high-grade subtype of TMEC, post-operative radiotherapy/chemotherapy is required due to its poor prognosis. However, the effectiveness of radiotherapy and chemotherapy is uncertain (2). Lymph node sampling or dissection is important during surgery to determine whether the tumor is confined to the lung. The patient's prognosis depends on the tumor subtype and whether the tumor is completely resected (2).

The aim of surgical resection is to completely remove the tumor with lymph node dissection while preserving as much of the lung parenchyma as possible (5). Depending on the extent of tumor invasion of the bronchi, there are two main types of bronchoplasty: wedge and sleeve (3). Reconstruction of the right secondary carina, a more complex bronchoplasty procedure, has rarely been reported (6, 7). Surgeons should have experience in simple or wedge bronchoplasty before performing sleeve or more complex bronchoplasty procedures (3).

Uniportal thoracoscopic surgery is nowadays a well-established and popular thoracic surgery. However, single-port VATS bronchoplasty, which requires experience in microscopic sewing, tying and suturing, is still a major technical challenge for the thoracic surgeon (8). In addition to the challenges of limited operative space and field inherent in single-port VATS, the undissected lung lobe structures limit field exposure and make it difficult to move and suture bronchial stumps (8). Several studies have confirmed that uniportal VATS bronchoplasty is safe and practical for use in selected patients and leads to acceptable outcomes (8, 9). In recent years, parenchymal sparing procedures (PSP) have been developed to reduce lung resection volume (4). Lucchi et al. have demonstrated that PSP achieves excellent functional and oncological outcomes in selected cases of low-grade bronchial neoplasms (4). They noted that the following points need to be met for parenchymal sparing bronchoplasty: 1. benign or low-grade malignant bronchial lesions without extrabronchial invasion; 2. small lesion base and normal peripheral bronchial tree; 3. no hilar or mediastinal lymph node metastases (4).

Interventional bronchoscopic therapy, as a simple and reproducible minimally invasive treatment modality, can control and relieve the symptoms of airway obstruction due to bronchial tumor (10). It has been suggested that bronchoscopic therapy has the potential to cure completely endobronchial low-grade malignancies confined to the bronchus (10). However, bronchoscopic treatment does not guarantee complete resection of the tumor, and Chen et al. reported local recurrence in 1/8 of low-grade TMEC and up to 2/3 of high-grade TMEC (10). In addition, bronchoscopic therapy does not allow for lymph node sampling or dissection; after all, lymph node metastases can occur in low-grade malignant tumors.

In this paper, we report the first case of single-port thoracoscopic parenchymal sparing right secondary carinal resection and reconstruction and systemic lymph node dissection for the treatment of TMEC. Obviously, a right upper lung sleeve resection is simpler and more likely to ensure negative margins. However, given the patient's 30+ year history of bronchial asthma, resection of a lung lobe would have meant a reduced quality of life. Tension-free suturing of the airway is important for bronchial reconstruction. Some authors recommend interrupted sutures to facilitate better size matching, less anastomotic ischaemia and to avoid suture loosening and winding. Considering the technical difficulty of using interrupted sutures under a single hole, a continuous suture minimizes the difficulty of suturing and also avoids the interference of visualization and suture manipulation caused by having multiple sutures in the incision at the same time. Therefore, we have adopted continuous sutures for bronchial anastomoses. In addition, the anterior bronchial wall is reinforced with interrupted sutures and the anterior mediastinal fatty tissue is freed to cover the anastomosis, further reducing the risk of postoperative bronchopleural fistula or anastomotic dehiscence (11).

In conclusion, this case highlights that uniportal video-assisted thoracoscopic parenchymal sparing secondary carinal resection and reconstruction for TMEC is safe and feasible under the hands of surgeon with extensive VATS experience. It can preserve lung function and result in excellent short-term outcomes. Moreover, further follow-up is required to assess the long-term outcomes of this patient.

## Data Availability Statement

The original contributions presented in the study are included in the article/Supplementary Material, further inquiries can be directed to the corresponding author.

## Ethics Statement

The studies involving human participants were reviewed and approved by the Clinical Research Ethics Committee of the Second Xiangya Hospital. The patients/participants provided their written informed consent to participate in this study.

## Author Contributions

YH drafted and edited this manuscript, assisted in the surgery, and analyzed patient data. XC, SR, CZ, LW, PX, and FY analyzed patient data. WL performed the surgery, edited this manuscript, and analyzed patient data. All authors read and approved the final manuscript.

## Funding

This work was supported by National Natural Science Foundation of China (81972638, 81972195, and 82172879), the Natural Science Foundation of Hunan Province, China (2019JJ30038), the Hunan Provincial Key Area R&D Program (2019SK2253), the Scientific Research Program of Hunan Provincial Health Commission (20201047), and the Clinical Medical Technology Innovation Guide Project of Hunan Province (S2020SFYLJS0311).

## Conflict of Interest

The authors declare that the research was conducted in the absence of any commercial or financial relationships that could be construed as a potential conflict of interest.

## Publisher's Note

All claims expressed in this article are solely those of the authors and do not necessarily represent those of their affiliated organizations, or those of the publisher, the editors and the reviewers. Any product that may be evaluated in this article, or claim that may be made by its manufacturer, is not guaranteed or endorsed by the publisher.
